# The Role of Mechanical Stimulation in Recovery of Bone Loss—High *versus* Low Magnitude and Frequency of Force

**DOI:** 10.3390/life4020117

**Published:** 2014-04-02

**Authors:** Mamta Patel Nagaraja, Hanjoong Jo

**Affiliations:** 1 National Aeronautics and Space Administration, Houston, TX 77058, USA; E-Mail: mamta.nagaraja@nasa.gov; 2 Wallace H. Coulter Department of Biomedical Engineering, Georgia Institute of Technology and Emory University, Health Sciences Research Building, E170, Atlanta, GA 30322, USA; 3 Department of Medicine, Division of Cardiology, Emory University, Atlanta, GA 30322, USA

**Keywords:** bone loss, astronauts, spaceflight, mechanical stimulation

## Abstract

Musculoskeletal pathologies associated with decreased bone mass, including osteoporosis and disuse-induced bone loss, affect millions of Americans annually. Microgravity-induced bone loss presents a similar concern for astronauts during space missions. Many pharmaceutical treatments have slowed osteoporosis, and recent data shows promise for countermeasures for bone loss observed in astronauts. Additionally, high magnitude and low frequency impact such as running has been recognized to increase bone and muscle mass under normal but not microgravity conditions. However, a low magnitude and high frequency (LMHF) mechanical load experienced in activities such as postural control, has also been shown to be anabolic to bone. While several clinical trials have demonstrated that LMHF mechanical loading normalizes bone loss *in vivo*, the target tissues and cells of the mechanical load and underlying mechanisms mediating the responses are unknown. In this review, we provide an overview of bone adaptation under a variety of loading profiles and the potential for a low magnitude loading as a way to counteract bone loss as experienced by astronauts.

## 1. Introduction

One of the primary functions of bone is providing mechanical integrity for both protection and locomotion. Bone adaptation is the inherent change in bone mass and architecture in response to strain induced by mechanical loads. Three rules govern bone adaptation as follows: (1) adaptation is driven by a dynamic stimulus, (2) adaptation requires only a relatively short duration of loading, and (3) bone cells become accustomed to routine mechanical loading [1]. The response of bone to loading or unloading is dependent on both genetic and epigenetic factors. While genetics outlines the general shape, length, and architecture, changes in mechanical environment elicit adaptive responses. According to Wolff’s law, bone architecture is defined by mathematical laws, which state that thickness, number, and distribution of trabeculae must correspond to distribution of mechanical stresses, and the trabeculae should be loaded axially in compression and tension [1,2]. It was later elucidated that strain resulting from mechanical stress itself could cause adaptive responses, and Frost defined a minimum effective strain that had be induced to instigate such a response [3]. Additionally, it was shown that bone responded to dynamic and not static strain [4]. These findings regarding bone adaptation have been formed into mathematical laws, calculating that a strain stimulus is a function of both strain magnitude and frequency. Since strain loading is dynamic, the strain stimulus can be defined using the Fourier method as shown in Equation (1):

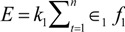
(1)
where E = strain stimulus, k = proportionality constant, ε = peak to peak strain magnitude, and f = frequency. This equation is critical in bone adaptation because it dictates that a static load would not cause a response since f = 0 and that adaptation is proportional to strain magnitude. However, it should be noted that this model predicts a linear relationship between strain magnitude and frequency, and it is well known that biology seldom encompasses precise linearity.

Strain is defined as a change in length relative to the object’s original length, and it is widely accepted that strain initiates mechanical force translation into a signal that can be recognized by cells active in bone metabolism [5]. Both the intensity and duration of the load play critical roles in defining adaptation to mechanical deformation. To avoid failure, the applied load must not induce strain beyond the bone tissue yield, and this level has been measured to be over 0.7% or 7000 microstrain (με) [5]. To determine functional strain levels in bone, strain gauges have been inserted *in vivo* in a host of animals, including dog, pig, turkey, sheep, and horse, and strain was measured during physical activity such as galloping or trotting. Remarkably, regardless of size, the maximum peak strain was measured to be within the range of 2000–3000 με for all animals, and this species-independent uniform peak strain is a concept called dynamic strain similarity [4].

In mechanical testing of bone, diverse loading conditions have been used to assess bone properties. Previous studies have shown that in cortical bone tissue, application of 2000 με at a frequency of 0.5 Hz maintained bone mass [6,7]. According to Equation (1), a similar response should be observed if the frequency was increased to 10 Hz and strain decreased to 100 με. This trend bound by Equation (1) was observed experimentally when the frequency was increased to 1 Hz and only a strain of 1000 με was needed to maintain bone mass. Further, at a frequency of 30 Hz, only 70 με was needed to inhibit bone resorption [7,8]. Thus, bone response to mechanical signals seems to correlate to increased frequency, meaning smaller strains induced by a lower force applied more frequently is ample to stimulate bone formation and maintain bone mass.

Bone adaptation occurs at both the macroscopic and microscopic levels, altering bone mass and architecture to maintain mechanical integrity for posture control and movement. It has been well accepted that activity with proper loading improves skeletal mass while disuse impedes it. It is also well known that muscle strength greatly impacts bone health as muscles constantly strain bone, causing adaptive responses within them. In the following sections, we review the accumulating evidence that exercise and muscle contraction both benefit the musculoskeletal system, despite delivering disparate signals to bone.

## 2. Mechanical Loading

### 2.1. Exercise: High Magnitude and Low Frequency

Osteopenia is a condition of decreased bone mass, and when bone mass has reduced to the point of a traumatic fracture risk, the condition is called osteoporosis. It is clear that there is an abundance of data suggesting that exercise promotes skeletal benefits, and the variation among studies is partially due to the type of exercise tested, patient population, and data analysis. However, there remains a common conclusion in the vast majority of studies that exercise provides skeletal benefit, albeit sometimes site-specific.

Exercise or physical activity beyond normal, daily routine exists in copious forms and induces distinct loads on the body. For instance, walking imposes a load of 1 g (1 times body weight) while running increases the load to 3–4 g, and jumping hurdles further augments the loading to 5 g [9]. Exercise can be regarded as a high magnitude (greater than 1g) and low frequency (1–2 Hz) repetitive force. The benefits of exercise have been inexorably tested, documented, and relayed to the general public, and it has been shown to increase bone and muscle mass [10,11,12]. The last two decades have provided insight into exercise intervention trials in the normal population, beyond that of comparing elite athletes to sedentary controls. The exercise regimes in these experiments ranged from aerobic *vs.* stretching control to high weight and low repetition resistance weight training *vs.* low weight and high repetition weight training. Subjects varied within a gamut of ages from a young 10 years to post-menopausal women over 50 years old. Studies continued for at least eight months and used bone mineral density (BMD) as a marker of skeletal improvement.

In one study involving young women ages 20–35, researchers investigated the use of exercise and calcium supplementation on peak bone mass. These women were divided into an exercise group consisting of weight training combined with aerobic activity or a stretching control group. After two years of exercise or control, there was a significant increase in BMD in the spine, femoral neck and trochanter, and calcaneus due to exercise, but calcium supplementation did not improve BMD in any tested location [13]. In another study with post-menopausal women, researchers explored the effects of resistance training (high load, low repetitions) *vs.* endurance training (low load, high repetitions) on bone mass in the forearm and hip. After one year of exercise intercession, there were significant increases in BMD at the femoral trochanter in the hip and the distal radius of the arm with resistance training while endurance training only improved mid-radius BMD. Using the one repeat maximum (1-RM) method, the researchers also found that muscle strength increased in both groups. The study concluded that the peak load was more important than the number of repetition cycles to increase bone mass in early post-menopausal women [14]. In another study of pre-menopausal women (age range of 28–39 years), subjects were assigned to exercise or non-exercise control groups and monitored for 1.5 years. The exercise group showed an increase in femur trochanter BMD by one year but no change in total, arm, or leg BMD compared to control. In addition, they used the 1-RM method to evaluate muscle strength and found a 58% increase in the exercise group when compared to baseline and no increase in the control group [15].

However, not all studies have reported increases in BMD at the femur, a site at which many fractures occur in osteoporotic patients. One study explored whether aerobic or weight training benefited the skeletal system in young college-aged women (mean age 19.9 years) over an eight month period. They found increases in spine lumbar BMD in both running and weight training groups compared to a non-exercise control group but no significant changes at the hip [11]. In another study evaluating the effects of jumping in pre- and post-menopausal women, the subjects were assigned to perform 50 vertical jumps six times per week. Mechanical loads induced to the joints were measured by ground reaction forces, amounting to 3 g/jump for pre-menopausal women and 4 g/jump for post-menopausal subjects. After five months, there was a 2.8% increase in BMD at the femur in the pre-menopausal group but no change in the post-menopausal women after one year or 1.5 years [16]. Thus, there was a clear benefit for younger women but not older, post-menopausal subjects.

The benefits of exercise have also been seen in disuse such as bed rest studies. For example, one study exposed an experimental group to 17 weeks of bed rest along with a regimen of resistive training [17]. Compared to a control group exposed to bed rest without exercise, the experimental group had greater bone mineral density in areas such as the hip, pelvis, and foot. Specific bone formation markers such as alkaline phosphatase and osteocalcin were also increased by exercise [17]. Further, recent data from the International Space Station (ISS) show partial inhibition of expected bone loss in astronauts when exercise was combined with the anti-resorptive drug alendronate [18]. In this study, astronauts started alendronate three weeks prior to flight and throughout the mission while also exercising on board the ISS. A key finding was attenuation of the expected bone loss in the hip, spine, and pelvis with resistive training [18]. The advanced resistive exercise device (ARED) on board the ISS is now capable of providing loads of 600 lbs, an upgrade from the interim resistive exercise device (iRED) that produced only 297 lbs [19]. The iRED provided little bone support, similar to aerobic stimulation. In a study comparing missions with access to the iRED or ARED, astronauts who used the ARED showed no change in pre- and post-flight bone mineral density measurements [19]. Thus, astronauts return to Earth showing significant steps towards successful bone remodeling through a combination of nutrition and exercise. However, the strength of the new bone compared to pre-flight bone remains unknown [19]. In a recent study evaluating 45 astronauts who had been in space from 4–6 months, bone loss was between 2% and 9% in areas such as the lumbar spine, trochanter, pelvis, and femoral neck. Further, 50% recovery of bone mineral density levels occurred within nine months after returning to Earth [20].

It is clear that there is an abundance of data suggesting that exercise promotes skeletal benefits, and the variation among the studies is probably partially due to the type of exercise tested. These discrepancies allow for appreciation of the complex nature of mechanical loading and subsequent bone adaptation. For example, during jumping, there is loading due to absorption of the impact as well as muscle-generated forces applied to the bone. In gymnasts, dismounting from the parallel bars stimulates an immense load amounting to approximately 11 g, which partially expounds why gymnasts encompass a hip BMD greater than other athletes [9,21]. However, in the Bassey study, jumping applied no more than 4 g reaction forces to the joints of the subjects, equivalent to forces from running [16]. Running has been generally shown to not improve femur BMD, and thus, in this study, the pre-menopausal women probably achieved increased femoral BMD because of strain induced by muscle-derived tension to the bone rather than impact [9]. However, with the complexity of mechanical loading and bone adaptation, there is most likely a host of other reasons as to why lower loads did not improve BMD in older women but did in younger women. We can only surmise conclusions from the clinical data available and continue to investigate other treatment options for bone loss.

It is generally well accepted that the growing skeleton is most likely to benefit from exercise as bone modeling and remodeling ensure optimal mechanical properties of bone, removing old, aged bone and replacing it with new, stronger bone. Exercise during childhood assists in the acquisition of bone as well as remodeling its architecture. It has been shown that tennis players who began training during childhood had increased BMD, bone mineral content, and cortical wall thickness compared to players who began playing during adulthood [22]. Furthermore, a study investigating the effects of exercise on pre-pubertal young boys (mean age 10.4 years) found that not only did the BMD of all boys increase over the course of the study as expected but also physical education intervention for eight months increased BMD twice that of controls in the exercise group. The researchers concluded based on all parameters measured that exercise before puberty may increase femur volumetric BMD by increasing cortical thickness [23].

Mounting data makes it quite lucid that exercise provides benefit to the musculoskeletal system, despite the variation among clinical trials. There is currently no defined exercise regime that seems to be best suited for improving bone mass, and if the field of research can adequately compare premium exercise routines for various age groups, this would be beneficial to the general public.

### 2.2. Muscle Contraction: Low Magnitude and High Frequency

As humans age, the harsh effects of high impact becomes a burden that the ailing mature skeleton can no longer efficiently tolerate. While several factors dictate osteoporosis, the pathology is exacerbated by increased age, coinciding with decreased muscle strength and posture stability. As ageing continues, there is a drastic degeneration in muscle strength (sarcopenia), leading to decreases in muscle-bone movement. Thus, the elderly population is prone to accidental falls and subsequent injury, leading to bone fractures. At each joint in the body, muscles apply forces to attached bones such that these signals are fired rapidly at low level movements that are imperceptible at the macroscopic level. Muscle contractions are constantly applied to the bone in everyday tasks such as maintaining posture while standing or sitting [24]. As muscle atrophies with age, these signals to the skeleton also diminish. The notion of stimulating bone formation or inhibiting bone resorption with low magnitude and high frequency (LMHF) signals in patients with musculoskeletal diseases such as cerebral palsy [25,26], osteopenia [7], spinal cord injury [27], age-related osteoporosis [28], and post-menopausal osteoporosis [29] is a new, non-invasive treatment. It is ideal for patients who cannot sustain high impact exercises such as the elderly and disabled. It has been shown through animal and clinical trials that a LMHF mechanical stimulation is anabolic to bone [26,29,30,31,32,33]. Moreover, this type of stimulation has been applied to a mouse model with diet-induced obesity. The LMHF stimulation recovered trabecular bone loss caused by obesity when compared to control mice [34]. Although not yet experimented in space, this potential osteoporosis countermeasure may provide benefit to the musculoskeletal system in a microgravity environment.

While loads from exercise induce strain levels of 2000–3000 με, muscle contractions inflict strains much lower in magnitude on the order of 10 με. With conventional thought, these strains would not be hypothesized to play a role in regulating bone adaptation, growth, or remodeling. However, as discussed earlier, equation 1 demonstrates that a lower peak-to-peak strain fired more frequently could provide a strain stimulus capable of inducing an adaptive response. Previous studies have been performed to log the strain history of bone in various animals, defining specific features such as peak magnitudes and frequencies over a specified time range [35]. It is classically thought that the peak strain magnitudes during high load activities have the largest effect on bone response, leading to many strain gage analyses of bone centered on maximum strains [36,37,38]. These peak strains have been shown to be non-uniformly located over the cortex of the bone [36], meaning not all bone cells sense the same strain. Moreover, these peak strains are only experienced for short periods of the day, leaving the majority of the strain history to be defined by other strain magnitudes. In fact, in a turkey model, the maximum peak strain movements lasted for a total of 2 min/day, consisting of wing flaps or body shakes. However, turkey bones are still well adapted and strong enough to support flight, suggesting that other portions of the strain history must be involved in bone adaptation [39].

The vast majority of the strain history of various animals encompasses low level strains from long periods of standing or sitting. In fact, standing intervention for 3 hours/day during bed rest prevented bone loss, suggesting that the effect of muscle contractions needed for postural stability aided in preventing bone loss [40]. Studies performed in sheep showed that low magnitude strains were experienced frequently in the range of 40 Hz. In another study examining the strain history in three distinct species, *in vivo* bone strains were recorded from the weight-bearing tibia of an adult dog, turkey, and sheep and non-weight bearing ulna of turkey. They found that over the course of 12–24 h, the turkey ulnae had strains of 1000 με once per day, 100 με about 100 times/day, and 5 με thousands of times per day. In the tibia, all three animals had one event of 1000 με, more events at 100με than the ulnae, and thousands of events at <10με. Animals were videotaped to correlate the type of movement to points in the strain history, and walking induced the maximum strain from −1500 to 1000 με in the turkey tibia at a frequency of 0.6 Hz but a continued strain history through 40 Hz. Standing caused strains of ±10 με at a frequency of 40 Hz [35].

These studies show that a dominant contributor to the strain history recordings is non-vigorous activity, where muscle contraction is required during activities such as standing. As such, the question is whether these low magnitude strains impact bone remodeling and adaptation. Subsequent investigations have shown the utility of these low level signals in bone adaptation in a host of subjects, preventing or normalizing bone loss. In growing BALB mice, 0.3 g mechanical loading at 45 Hz induced strain oscillations of 10 με on the periosteal surface of the tibia as measured by *in vivo* strain gages. After treatment for three weeks for 15 min/day, there was a decrease in osteoclast activity compared to age-matched controls while bone formation rates (BFR) in the trabecular bone and mid-diaphyseal cortical bone were unchanged [41]. However, BFR in the endocortical surface of the metaphysis was increased in this study [41] while cortical bone has also been shown to not respond to these signals in other studies [30]. Total body mass, bone length, and matrix composition were not negatively altered by the LMHF load [41]. In another study, the left tibias of wildtype C57BL/6J mice were exposed to 0.3 g or 0.6 g loading at 45 Hz for 10 min/day while the right tibia acted as an internal control. Strain levels were as low as 3 με, and after three weeks of 0.3 g or 0.6 g loading, there was an increase in trabecular bone formation rate in the metaphysis. Bone morphology in the epiphysis was altered by increased cortical area and thickness [42]. Furthermore, the effects of LMHF loading on bone adaptation caused by disuse were investigated using the hindlimb unloading (HLU) model. Adult BALB mice were exposed to 0.3g load at 45 Hz for 10 min/day for a total of four or 21 days. There was a decrease in BFR due to HLU and an increase due to LMHF loading after 21 days treatment in both trabecular and cortical surfaces. After four days, there were decreases in gene expression for several genes, including type 1 collagen, osterix, matrix metalloproteinase protein 2 (MMP2), and osteonectin due to HLU. There was no change in expression at four days due to LMHF loading; however, after 21 days, there were increases in inducible nitric oxide synthase, MMP2, receptor activator of the nuclear factor κB, and type I collagen. There were no changes due to HLU or LMHF loading in cathepsin K, runt homology domain transcription factor 2, or MMP9 gene expression after four or 21 days [33].

Studies involving LMHF loading then advanced to rat models, and in one study, adult rats were subjected to control, LMHF mechanical stimulation, disuse by HLU, HLU intervened by LMHF loading for 10 min/day, or HLU intervened with normal weight bearing for 10 min/day. After 28 days, there was an increase in bone formation rate in the proximal tibia with exposure to LMHF loading. HLU inhibited BFR while intervention with normal weight bearing slightly increased bone formation. However, LMHF loading normalized bone formation rate in the HLU group compared to age-matched controls [32]. There have also been investigations in larger animal models such as turkey and sheep. In one study, the hindlimbs of adult sheep were exposed to approximately 5 με induced by 0.3 g loading for 20 min/day. After one year of treatment, trabecular bone were evaluated with micro-computed tomography and mechanically tested. Mechanical loading increased mineral content and trabecular number while trabecular spacing decreased, displaying increased trabecular quantity and thickening. There was an increase in stiffness and mechanical strength in the longitudinal direction of weight bearing. This study was critical because it showed that not only does LMHF loading increase bone mass but also the quality of the trabecular bone, and these effects were observed in a larger animal at a considerably longer time point than previous findings [31].

While animal studies were imperative in moving toward testing this device in humans, the most critical information supporting the hypothesis that such low magnitude mechanical signals can induce bone mass and adaptation were obtained as studies advanced to humans. LMHF mechanical loading has been shown to benefit various groups, including children with disabilities, young women with low bone mineral density (BMD), and post-menopausal women with osteoporosis. In a pilot clinical trial, children with disabilities affecting muscular strength such as cerebral palsy and muscular dystrophy were treated with mechanical loading at 0.3 g at 90 Hz for 10 min/day for 5 days/week for a total of six months. There was a 6.3% increase in tibia volumetric trabecular bone mineral density (vTBMD) with treatment compared to placebo control after brief exposure to LMHF loading, which was remarkable since compliance in this study was fairly low at 44% or about 4.4 min/day of treatment. However, there was no effect on vTBMD in the spine [26]. The disabilities endured by these children results in poor muscular strength and limited mobility. Therefore, it is possible that these physiological signals of muscle contraction to the bone are also diminished in these patients. Since LMHF loading mimics the signals outputted by the musculature to the bone, it is possible that the children had increased BMD, at least in specific sites, because the diminished muscular signals were replaced by the LMHF loading device.

There have also been clinical trials investigating the effects of LMHF loading on young women, and in one study, 48 women (age range of 15–20 years) with low BMD and a history of at least one skeletal fracture were exposed to either control or treatment conditions for 10 min/day at 0.3 g and 30 Hz for one year. There was an increase in trabecular bone in the lumbar vertebrae and cortical bone of the femur midshaft compared to controls. Moreover, there was an increase in muscle cross section area, and these beneficial results were dependent on compliance level [43]. Furthermore, clinical trials in post-menopausal women have been performed, and in one study, treatment at 0.2 g at 30 Hz for two 10 min/day treatments for one year increased BMD in the femur compared to placebo control. The women in the placebo control group lost 2% BMD over the experimental time frame. There was less bone loss in the spine in the treated group compared to controls, and these effects were dependent on compliance. As such, those who complied with the treatment protocol most stringently had the greatest beneficial effects [29].

Thus, LMHF mechanical loading has been shown to benefit the musculoskeletal system despite some variation among studies. The device has recently been approved for treatment of osteoporosis, but long term studies will provide the most beneficial information as to its safety and efficacy in preventing or normalizing bone loss.

## 3. Bone Loss Recovery Potential

Cultured osteoblasts can directly sense and respond to an extremely low magnitude mechanical stimulus when applied at a high frequency, leading to osteogenic changes at least *in vitro* [44]. This study suggests that osteoblasts are partially responsible for the anabolic effects of LMHF loading observed *in vivo* in both animals and humans [25,26,32]. These results are significant because they suggest that low loading in intact animals and humans may directly stimulate osteoblasts and stimulate bone formation responses. It is widely accepted that bone is responsive to signals that create peak strain magnitudes of 2000–3500 microstrains (με), such as those created from physical activities like running [4,7,45]. However, muscle contractions during standing impose strains in the spectral range of 10–50 Hz of at least two orders of magnitude lower than high loads or strenuous activities [7] and have recently been shown to normalize bone loss [29,31,32]. Previous studies have shown that in cortical bone tissue, application of 2000 με at a frequency of 0.5 Hz (high magnitude and low frequency) maintained bone mass [6,7]. When the frequency was increased to 1 Hz, only a strain of 1000 με was needed to maintain bone mass, and at 30 Hz, only 70 με (low magnitude, high frequency) was needed to inhibit bone resorption [7,8]. Thus, bone response to mechanical loading appears to correlate with the product of frequency and load magnitude, meaning small strains induced by a low force could stimulate bone formation and maintain bone mass if applied at a high frequency. However, the underlying molecular mechanisms regulating how such a low level signal can be anabolic to bone tissue and which cells or tissues sense and mediate the response are unknown. While there is considerably less data, there is evidence that vibration also affects osteocytic cells [46]. These studies show that the effect of vibration on osteogenic cells is directly due to the LMHF stimulus and not a secondary effect of fluid shear [47,48]. Additionally, the LMHF signal must be used with caution as prolonged exposure to LMHF signals may be a pathogen to various physiological systems [49].

To examine the underlying mechanisms of the observed *in vivo* anabolic effects in response to LMHF loading, this study used a simulator of microgravity called the Random Positioning Machine (RPM) to induce a bone loss response *in vitro* using 2T3 cells [44,50,51]. As expected, the RPM decreased ALP activity and osteoblast mineralization and LMHF treatment increased both markers in control samples [44]. Moreover, LMHF prevented the RPM inhibition of ALP activity and mineralization, preventing bone loss responses induced by the simulated microgravity conditions. The effects of both the RPM and LMHF loading were much more dramatic on mineralization than ALP activity of 2T3 cells. This may be because ALP activity is an early indicator of bone formation and is transient [52], making it a less sensitive marker of osteogenesis, especially in response to mechanical stimuli. These findings show that osteoblasts may directly respond to the LMHF signal at least *in vitro* by induction of osteogenic markers, suggesting that bone may respond to LMHF signals *in vivo* through osteoblast cells.

Additionally, another study proposed a role for the nucleus in perceiving acceleration signals. The study investigated vibration levels with a frequency range of 5–100 Hz and measured various cell signaling markers of bone adaptation to mechanical loading. Particularly, nitric oxide correlated positively and prostaglandin E2 correlated negatively with the maximum acceleration rate of the vibration loads. The study concluded that these trends support the occurrence of nucleus oscillations, which suggests a physical basis for mechanotransduction of high-frequency loads [53].

Further, a recent study incorporated LMHF loading into a bed rest study, which is currently the only human analog to spaceflight’s microgravity conditions. In this study, the subjects were exposed to 90 days of bed rest. The experimental group was exposed to 10 min per day of LMHF stimulation at 0.3 or 0.5 g. The goal of the study was to investigate intervertebral disc response to the LMHF exposure as astronauts in spaceflight have shown bone loss in the spine. The results of this study showed that the stimulation mitigated disc morphology changes such as intervertebral swelling as observed by computed tomography [54]. A follow up study showed that addition of LMHF signals mitigated the deterioration of the intervertebral disc using hindlimb suspended animals, whereas allowing the animals to walk over ground alone was not sufficient [55]. This finding further demonstrates the preventive role of LMHF in bone loss of the intervertebral discs.

These studies show that osteoblasts may respond directly to a LMHF mechanical load and that the human body exposed to a simulated microgravity environment may benefit from LMHF loading [44,54]. It had not been previously reported how the LMHF may be sensed and transmitted in to the cells to induce the osteogenic responses. Potential mechanotransduction pathways may involve integrins [56], stretch-activated channels and the ensuing influx of extracellular calcium [56], or cell deformations and cytoskeleton [57,58]. Elucidating the mechanosensors and mechanotransduction pathways would be interesting future work. These pathways would further our understanding of bone physiology and the etiology of bone pathologies [59,60].

## 4. Conclusions and Perspectives

Musculoskeletal pathologies affect millions of people worldwide, and these studies reviewed here shed insight into their mechanisms of action. This information is vital to continue to develop potential therapies that may target the right signaling pathway or the correct gene that may provide a better treatment than what is available today. While high magnitude and low frequency loads such as running has been long recognized to increase bone and muscle mass under normal conditions, there is some data that shows a low magnitude and high frequency (LMHF) mechanical load experienced in activities such as postural control to be anabolic to bone. Future studies must continue to elucidate the role of LMHF loading in musculoskeletal etiologies. It is incumbent upon the musculoskeletal field to investigate various types of loading profiles and their impact on recovering negative skeletal changes.

## References

[1] Turner C.H. (1998). Three rules for bone adaptation to mechanical stimuli. Bone.

[2] Wolff J. (1892). Das Gesetz der Transformation der Knochen.

[3] Frost H.M. (1964). The Laws of Bone Structure.

[4] Rubin C.T., Lanyon L.E. (1984). Dynamic strain similarity in vertebrates; an alternative to allometric limb bone scaling. J Theor. Biol..

[5] Marcus R., Feldman D., Kelsey J.L. (2001). Osteoporosis.

[6] Rubin C.T., Lanyon L.E. (1984). Regulation of bone formation by applied dynamic loads. J. Bone Joint Surg. Am..

[7] Rubin C.T., Sommerfeldt D.W., Judex S., Qin Y. (2001). Inhibition of osteopenia by low magnitude, high-frequency mechanical stimuli. Drug Discov. Today.

[8] Rubin C.T., Lanyon L.E. (1985). Regulation of bone mass by mechanical strain magnitude. Calcif. Tissue Int..

[9] Marcus R. (1998). Exercise: Moving in the right direction. J. Bone Miner. Res..

[10] Vuori I. (1995). Exercise and physical health: Musculoskeletal health and functional capabilities. Res. Q. Exerc. Sport.

[11] Snow-Harter C., Bouxsein M.L., Lewis B.T., Carter D.R., Marcus R. (1992). Effects of resistance and endurance exercise on bone mineral status of young women: A randomized exercise intervention trial. J. Bone Miner. Res..

[12] Galloway M.T., Jokl P. (2000). Aging successfully: The importance of physical activity in maintaining health and function. J. Am. Acad. Orthop. Surg..

[13] Friedlander A.L., Genant H.K., Sadowsky S., Byl N.N., Gluer C.C. (1995). A two-year program of aerobics and weight training enhances bone mineral density of young women. J. Bone Miner. Res..

[14] Kerr D., Morton A., Dick I., Prince R. (1996). Exercise effects on bone mass in postmenopausal women are site-specific and load-dependent. J. Bone Miner. Res..

[15] Lohman T., Going S., Pamenter R., Hall M., Boyden T., Houtkooper L., Ritenbaugh C., Bare L., Hill A., Aickin M. (1995). Effects of resistance training on regional and total bone mineral density in premenopausal women: A randomized prospective study. J. Bone Miner. Res..

[16] Bassey E.J., Rothwell M.C., Littlewood J.J., Pye D.W. (1998). Pre- and postmenopausal women have different bone mineral density responses to the same high-impact exercise. J. Bone Miner. Res..

[17] Shackelford L.C., LeBlanc A.D., Driscoll T.B., Evans H.J., Rianon N.J., Smith S.M., Spector E., Feeback D.L., Lai D. (2004). Resistance exercise as a countermeasure to disuse-induced bone loss. J. Appl. Physiol..

[18] Leblanc A., Matsumoto T., Jones J., Shapiro J., Lang T., Shackelford L., Smith S.M., Evans H., Spector E., Ploutz-Snyder R. (2013). Bisphosphonates as a supplement to exercise to protect bone during long-duration spaceflight. Osteoporos. Int..

[19] Smith S.M., Heer M.A., Shackelford L.C., Sibonga J.D., Ploutz-Snyder L., Zwart S.R. (2012). Benefits for bone from resistance exercise and nutrition in long-duration spaceflight: Evidence from biochemistry and densitometry. J. Bone Miner. Res..

[20] Sibonga J.D., Iwaniec U.T., Shogren K.L., Rosen C.J., Turner R.T. (2007). Effects of parathyroid hormone (1–34) on tibia in an adult rat model for chronic alcohol abuse. Bone.

[21] Robinson T.L., Snow-Harter C., Taaffe D.R., Gillis D., Shaw J., Marcus R. (1995). Gymnasts exhibit higher bone mass than runners despite similar prevalence of amenorrhea and oligomenorrhea. J. Bone Miner. Res..

[22] Haapasalo H., Sievanen H., Kannus P., Heinonen A., Oja P., Vuori I. (1996). Dimensions and estimated mechanical characteristics of the humerus after long-term tennis loading. J. Bone Miner. Res..

[23] Bradney M., Pearce G., Naughton G., Sullivan C., Bass S., Beck T., Carlson J., Seeman E. (1998). Moderate exercise during growth in prepubertal boys: Changes in bone mass, size, volumetric density, and bone strength: A controlled prospective study. J. Bone Miner. Res..

[24] Rubin C., Turner A.S., Bain S., Mallinckrodt C., McLeod K. (2001). Anabolism. Low mechanical signals strengthen long bones. Nature.

[25] Gray B., Hsu J.D., Furumasu J. (1992). Fractures caused by falling from a wheelchair in patients with neuromuscular disease. Dev. Med. Child Neurol..

[26] Ward K., Alsop C., Caulton J., Rubin C., Adams J., Mughal Z. (2004). Low magnitude mechanical loading is osteogenic in children with disabling conditions. J. Bone Miner. Res..

[27] Asselin P., Spungen A.M., Muir J.W., Rubin C.T., Bauman W.A. (2011). Transmission of low-intensity vibration through the axial skeleton of persons with spinal cord injury as a potential intervention for preservation of bone quantity and quality. J. Spinal Cord Med..

[28] Kiel D.P., Hannan M.T., Barton B.A., Bouxsein M.L., Lang T.F., Brown K.M., Shane E., Magaziner J., Zimmerman S., Rubin C.T. (2010). Insights from the conduct of a device trial in older persons: Low magnitude mechanical stimulation for musculoskeletal health. Clin. Trials.

[29] Rubin C., Recker R., Cullen D., Ryaby J., McCabe J., McLeod K. (2004). Pevention of postmenopausal bone loss by a low-magnitude, high-frequency mechanical stimuli: A clinical trial assessing compliance; efficacy; and safety. J. Bone Miner. Res..

[30] Rubin C., Turner A.S., Mallinckrodt C., Jerome C., McLeod K., Bain S. (2002). Mechanical strain, induced noninvasively in the high-frequency domain, is anabolic to cancellous bone, but not cortical bone. Bone.

[31] Rubin C., Turner A.S., Muller R., Mittra E., McLeod K., Lin W., Qin Y.X. (2002). Quantity and quality of trabecular bone in the femur are enhanced by a strongly anabolic, noninvasive mechanical intervention. J. Bone Miner. Res..

[32] Rubin C., Xu G., Judex S. (2001). The anabolic activity of bone tissue, suppressed by disuse, is normalized by brief exposure to extremely low-magnitude mechanical stimuli. FASEB J..

[33] Judex S., Zhong N., Squire M.E., Ye K., Donahue L.R., Hadjiargyrou M., Rubin C.T. (2005). Mechanical modulation of molecular signals which regulate anabolic and catabolic activity in bone tissue. J. Cell. Biochem..

[34] Chan M.E., Adler B.J., Green D.E., Rubin C.T. (2012). Bone structure and B-cell populations, crippled by obesity, are partially rescued by brief daily exposure to low-magnitude mechanical signals. FASEB J..

[35] Fritton S.P., McLeod K.J., Rubin C.T. (2000). Quantifying the strain history of bone: Spatial uniformity and self-similarity of low-magnitude strains. J. Biomech..

[36] Rubin C.T., Lanyon L.E. (1982). Limb mechanics as a function of speed and gait: A study of functional strains in the radius and tibia of horse and dog. J. Exp. Biol..

[37] Lanyon L.E., Smith R.N. (1970). Bone strain in the tibia during normal quadrupedal locomotion. Acta Orthop. Scand..

[38] Carter D.R., Smith D.J., Spengler D.M., Daly C.H., Frankel V.H. (1980). Measurement and analysis of *in vivo* bone strains on the canine radius and ulna. J. Biomech..

[39] Adams D.J., Spirt A.A., Brown T.D., Fritton S.P., Rubin C.T., Brand R.A. (1997). Testing the daily stress stimulus theory of bone adaptation with natural and experimentally controlled strain histories. J. Biomech..

[40] Issekutz B., Blizzard J.J., Birkhead N.C., Rodahl K. (1966). Effect of prolonged bed rest on urinary calcium output. J. Appl. Physiol..

[41] Xie L., Jacobson J.M., Choi E.S., Busa B., Donahue L.R., Miller L.M., Rubin C.T., Judex S. (2006). Low-level mechanical vibrations can influence bone resorption and bone formation in the growing skeleton. Bone.

[42] Garman R., Gaudette G., Donahue L.R., Rubin C., Judex S. (2007). Low-level accelerations applied in the absence of weight bearing can enhance trabecular bone formation. J. Orthop. Res..

[43] Gilsanz V., Wren T.A., Sanchez M., Dorey F., Judex S., Rubin C. (2006). Low-level, high-frequency mechanical signals enhance musculoskeletal development of young women with low BMD. J. Bone Miner. Res..

[44] Patel M.J., Chang K.H., Sykes M.C., Talish R., Rubin C., Jo H. (2009). Low magnitude and high frequency mechanical loading prevents decreased bone formation responses of 2T3 preosteoblasts. J. Cell. Biochem..

[45] Murfee W.L., Hammett L.A., Evans C., Xie L., Squire M., Rubin C., Judex S., Skalak T.C. (2005). High-frequency, low-magnitude vibrations suppress the number of blood vessels per muscle fiber in mouse soleus muscle. J. Appl. Physiol..

[46] Lau E., Al-Dujaili S., Guenther A., Liu D., Wang L., You L. (2010). Effect of low-magnitude, high-frequency vibration on osteocytes in the regulation of osteoclasts. Bone.

[47] Uzer G., Manske S.L., Chan M.E., Chiang F.P., Rubin C.T., Frame M.D., Judex S. (2012). Separating Fluid Shear Stress from Acceleration during Vibrations *in Vitro*: Identification of Mechanical Signals Modulating the Cellular Response. Cell. Mol. Bioeng..

[48] Uzer G., Pongkitwitoon S., Ete Chan M., Judex S. (2013). Vibration induced osteogenic commitment of mesenchymal stem cells is enhanced by cytoskeletal remodeling but not fluid shear. J. Biomech..

[49] Chan M.E., Uzer G., Rubin C.T. (2013). The potential benefits and inherent risks of vibration as a non-drug therapy for the prevention and treatment of osteoporosis. Curr. Osteoporos. Rep..

[50] Huijser R.H. (2000). Desktop RPM: New Small Size Microgravity Simulator for the Bioscience Laboratory.

[51] Pardo S.J., Patel M.J., Sykes M.C., Platt M.O., Boyd N.L., Sorescu G.P., Xu M., van Loon J.J.W.A., Wang M.D., Jo H. (2005). Simulated microgravity using the Random Positioning Machine inhibits differentiation and alters gene expression profiles of 2T3 preosteoblasts. Am. J. Physiol. Cell Physiol..

[52] Lian J.B., Stein G.S. (1995). Development of the osteoblast phenotype: Molecular mechanisms mediating osteoblast growth and differentiation. Iowa Orthop. J..

[53] Bacabac R.G., Smit T.H., van Loon J.J., Doulabi B.Z., Helder M., Klein-Nulend J. (2006). Bone cell responses to high-frequency vibration stress: Does the nucleus oscillate within the cytoplasm?. FASEB J..

[54] Holguin N., Muir J., Rubin C., Judex S. (2009). Short applications of very low-magnitude vibrations attenuate expansion of the intervertebral disc during extended bed rest. Spine J..

[55] Holguin N., Uzer G., Chiang F.P., Rubin C., Judex S. (2011). Brief daily exposure to low-intensity vibration mitigates the degradation of the intervertebral disc in a frequency-specific manner. J. Appl. Physiol..

[56] Iqbal J., Zaidi M. (2005). Molecular regulation of mechanotransduction. Biochem. Biophys. Res. Commun..

[57] Ingber D. (1999). How cells (might) sense microgravity. FASEB J..

[58] Ingber D.E. (2006). Cellular mechanotransduction: Putting all the pieces together again. FASEB J..

[59] Thompson W.R., Rubin C.T., Rubin J. (2012). Mechanical regulation of signaling pathways in bone. Gene.

[60] Ozcivici E., Luu Y.K., Adler B., Qin Y.X., Rubin J., Judex S., Rubin C.T. (2010). Mechanical signals as anabolic agents in bone. Nat. Rev. Rheumatol..

